# The Relationship Between Physical Activity and Depression in College Students: A Systematic Review and Meta-Analysis

**DOI:** 10.3390/brainsci15080875

**Published:** 2025-08-18

**Authors:** Xiaorui Huang, Zhuying Chen, Ze Xu, Xiaojie Liu, Yuanyuan Lv, Laikang Yu

**Affiliations:** 1 Beijing Key Laboratory of Sports Performance and Skill Assessment, Beijing Sport University, Beijing 100084, China; 13115856760@163.com (X.H.); sunflowerlyy@bsu.edu.cn (Y.L.); 2 Sports Coaching College, Beijing Sport University, Beijing 100084, China; 3 Department of Strength and Conditioning Assessment and Monitoring, Beijing Sport University, Beijing 100084, China; 2023210126@bsu.edu.cn (Z.C.); xz2000413@163.com (Z.X.); 4 Department of Pharmacology and Toxicology, Medical College of Wisconsin, Milwaukee, WI 53226, USA; xiaojieliu@mcw.edu; 5 China Institute of Sport and Health Science, Beijing Sport University, Beijing 100084, China

**Keywords:** physical activity, depression, college students, systematic review, meta-analysis

## Abstract

**Objectives**: Depression is a significant and growing global concern with substantial societal impact. College students, being particularly vulnerable to depression, necessitate exploration of physical activity (PA) as a potential mitigating factor. This study aims to examine the relationship between PA and depression in college students. **Methods**: Studies were identified through systematic searches of PubMed, Embase, Cochrane, Scopus, and Web of Science. The Pearson correlation coefficient was utilized to assess the PA–depression relationship. Heterogeneity was evaluated, and subgroup analyses were performed. Sensitivity analysis via the leave-one-out method was conducted. Quality assessment was assessed using the Joanna Briggs Institute literature quality assessment approach, resulting in the inclusion of 38 high-quality, low-risk studies. **Results**: A significant negative correlation between PA and depression was found (r = −0.238; 95% CI, −0.307 to −0.173; *p* < 0.001). Subgroup analyses revealed notable PA–depression correlations post-COVID-19 (r = −0.324; 95% CI, −0.493 to −0.132; *p* < 0.001), in developing countries (r = −0.202; 95% CI, −0.213 to −0.191; *p* < 0.001), and in physical education majors (r = −0.390; 95% CI, −0.589 to −0.147; *p* < 0.001). Moderate PA levels were associated with reduced depression (r = −0.428; 95% CI, −0.708 to −0.031; *p* = 0.035). **Conclusions**: This systematic review and meta-analysis suggests that PA is significantly and negatively associated with depression and plays a crucial role in alleviating depression in college students. Various influences, including the pandemic, national development level, student major, and PA intensity, moderate this relationship. Post-pandemic, developing countries, physical education majors, and moderate PA intensity emerged as optimal factors for enhancing the depression-alleviating effects of PA.

## 1. Introduction

University students, transitioning from adolescence to adulthood, are particularly vulnerable to emotional challenges that may lead to depressive symptoms and problematic behaviors [1]. Depression, a complex and heterogeneous condition extending beyond low mood, presents with diverse clinical manifestations and long-term impacts often intersecting with other conditions [2]. This complexity makes depression a difficult emotional state to fully understand [3]. Major depression, a prevalent condition, is a significant risk factor for cardiovascular morbidity and mortality [4,5]. Academic and employment pressures significantly contribute to the heightened risk of depression in college students [6,7]. Research indicates that childhood trauma, combined with academic stress, can increase the likelihood of depression in college students [8]. Despite being a socially advantaged group, college students face an elevated risk of depression exceeding that of the general population [9], which can impair academic performance and reduce scholarly output [10].

Mainstream depression treatments encompass pharmacological interventions, evidence-based psychological therapies such as cognitive behavioral therapy (CBT), and somatic non-pharmacological therapy [11]. Exercise is also recommended as a low-risk adjunctive therapy for depression [12,13,14,15]. However, depression remains challenging to fully resolve, with only 28% of individuals achieving complete remission post-treatment [16]. Prevention strategies are more effective in reducing the burden of depression than focusing solely on post-onset interventions [17]. Taliaferro et al. [18] demonstrated that depression can trigger suicidal ideation in college students, causing substantial psychological harm. Scholarly debate continues regarding the efficacy of physical activity (PA) in mitigating depression risks in this cohort. While some studies suggest PA reduces depression likelihood by inducing neurobiological changes and enhancing brain function [19], others propose no clear link or even a positive association between PA and depressive symptoms [20,21]. This uncertainty underscores the need for further research.

Previous meta-analyses have predominantly focused on general population [1,22,23], children [24,25,26], pregnant women [27,28], and older adults [29], with limited evidence synthesis for college students, a population undergoing critical psychological development marked by academic stress, identity transition, and social reconstruction. Only one meta-analysis has addressed common mental health problems in college students, including depression, anxiety, obsessive–compulsive disorder (OCD), and post-traumatic stress disorder (PTSD) [30]. However, it did not elucidate the specific mechanisms linking PA to depressive symptoms. The coronavirus disease 2019 (COVID-19) pandemic has introduced unique social isolation and uncertainty, significantly impacting college students’ mental health. While a prior study has explored PA’s role in mitigating depression during the pandemic [31], no meta-analysis has synthesized post-pandemic evidence for this population.

Therefore, this pioneering meta-analysis investigates the relationship between PA and depressive symptoms in college students. Considering the potential moderating effects of PA intensities, academic disciplines, cultural backgrounds, and pre-/post-pandemic temporal contexts on observed outcomes, this study employs stratified analysis to delineate the influence of these variables.

## 2. Materials and Methods

The research protocol was registered in PROSPERO (CRD420251009881) and adhered to the Preferred Reporting Items for Systematic Reviews and Meta-Analyses guidelines (PRISMA, 2020) [32].

### 2.1. Search Strategy

Using a predefined search strategy, we systematically retrieved studies from the PubMed, Embase, Cochrane, Scopus, and Web of Science. The search strategy combined subject headings and free-text terms, with intra-category terms linked by “OR” and inter-category terms by “AND” (Table S1). The search concluded on 26 February 2025, and was limited to English-language studies. Relevant reviews and citations from the included studies were examined to identify additional eligible articles. Two independent reviewers (X.H. and Z.C.) screened titles, abstracts, and full texts for eligibility, with discrepancies resolved by a third reviewer (L.Y.).

### 2.2. Eligibility Criteria

Study eligibility was defined according to the Population, Exposure, Comparison, Outcome (PECO) framework, with the following operational criteria: (a) Population: collegiate populations encompassing undergraduate, postgraduate, and professional degree students; (b) Exposure: PA quantitatively assessed through psychometrically validated instruments or explicitly reported parameters (modality, frequency, intensity, duration); (c) Comparison: observational analyses examining associations between PA exposure and depression outcomes; (d) Outcome: depressive symptomatology quantified as the primary clinical endpoint.

Exclusion criteria comprised (1) studies involving non-college populations; (2) investigations lacking a primary focus on PA–depression relationships; (3) absence of statistical evidence supporting PA–depression associations; (4) non-empirical research (e.g., reviews, commentaries). Crucially, all experimental designs, including randomized controlled trials (RCTs) and non-RCTs, were systematically excluded during screening to maintain methodological alignment with observational evidence synthesis objectives.

### 2.3. Data Extraction

Two reviewers (X.H. and Z.C.) independently extracted data using a standardized form, capturing (1) study details (title, author, year); (2) sample characteristics (size, gender distribution, age, academic grade, major); and (3) correlation coefficients (r-values) quantifying the PA–depression relationship. Discrepancies were resolved through third-party consultation.

### 2.4. Quality Assessment

The methodological quality of included studies was assessed using the Joanna Briggs Institute (JBI) Critical Appraisal Checklist, comprising 10 domains [33]. These domains encompass research purpose and theoretical foundation, participant selection criteria, inclusion/exclusion criteria, characterization of sample demographics, reliability and validity of data collection tools, measures to ensure data authenticity, ethical considerations, appropriateness of statistical methods, accuracy of results presentation, and articulation of research significance. Each domain was scored 0–2 (0: not addressed; 1: partially described; 2: comprehensively described), yielding a maximum total score of 20. Studies were categorized into high quality (≥16 points, indicating comprehensive coverage of critical methodological aspects), moderate quality (10–15 points, indicating some methodological deficiencies), and low quality (≤9 points, lacking essential methodological details). Two independent reviewers (X.H. and Z.C.) conducted evaluations, resolving discrepancies through consensus or third-party arbitration. The JBI framework offers a comprehensive evaluation system for various study designs, providing a standardized approach to assessing methodological transparency.

### 2.5. Statistical Analysis

The association between PA and depression risk was assessed using correlation coefficients (r) with 95% confidence intervals (CIs). Comprehensive meta-analysis software (Version 3; Biostat Inc., Englewood, NJ, USA) was used for quantitative synthesis. The “correlation and sample size” module analyzed bivariate correlation coefficients (r-values) and sample sizes from eligible studies. Pooled effect estimates were calculated using inverse-variance weighting, and 95% CIs determined via DerSimonian–Laird random-effects models to address heterogeneity. Cochran’s Q test and I^2^ statistic > 50% guided the choice of a random-effects meta-analysis framework [34]. Sensitivity analysis was performed using the leave-one-out method, and publication bias was assessed via Egger’s test and funnel plot inspection.

Subgroup analyses were conducted to examine potential effect modifiers across four key dimensions: (1) temporal context relative to the COVID-19 pandemic (pre-pandemic, peri-pandemic, post-pandemic), (2) national economic development status (developed countries, developing countries), (3) academic discipline affiliation (non-physical education majors, physical education majors), and (4) PA intensity levels [low physical activity (LPA), moderate physical activity (MPA), moderate-to-vigorous physical activity (MVPA), vigorous physical activity (VPA)].

## 3. Results

### 3.1. Study Selection

**Figure 1:**
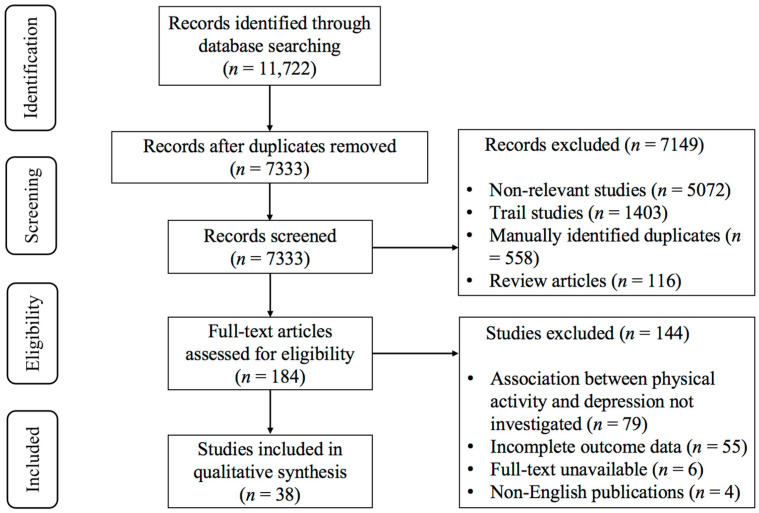
PRISMA flowchart of study selection.

An initial systematic search of electronic databases identified 11,722 potentially relevant records. After removing duplicates, 7333 unique records remained. Two independent researchers (X.H. and Z.C.) excluded 7149 records through blinded title and abstract screening based on predefined criteria, resulting in a full-text assessment of 184 articles. After full-text assessment, 144 studies were excluded for the following reasons (Table S2): (1) association between physical activity and depression not investigated (*n* = 79); (2) Incomplete outcome data (*n* = 55); (3) full-text unavailable (*n* = 6); and (4) non-English publications (*n* = 4). Finally, 38 studies met the inclusion criteria and were included in the systematic review and meta-analysis. The study selection process is illustrated in Figure 1.

### 3.2. Characteristics of the Included Studies

The included studies involved a total of 28,540 college students. Detailed demographic information for each study is provided in Table S3. The research spanned fifteen countries across four continents, with the majority conducted in Asia (26 studies), particularly in China (*n* = 19) [35,36,37,38,39,40,41,42,43,44,45,46,47,48,49,50,51,52,53], followed by Iran (*n* = 2) [54,55], South Korea (*n* = 2) [56,57], Japan (*n* = 2) [21,58], and The United Arab Emirates (*n* = 1) [59]. Additional studies were conducted in the Americas (6 studies; United States: *n* = 3, Peru: *n* = 1, Brazil: *n* = 1, Paraguay: *n* = 1) [18,60,61,62,63,64], Europe (5 studies; Germany: *n* = 2; Spain: *n* = 1, Switzerland: *n* = 1, Russia: *n* = 1) [20,65,66,67,68], and Africa (2 studies; Nigeria: *n* = 1, Egypt: *n* = 1) [59,69]. In the temporal dimension, studies were categorized into three phases: pre-COVID-19 [18,20,21,40,53,55,56,58,65,66,68], during COVID-19 [7,36,38,39,41,42,43,44,45,46,47,50,51,52,57,59,60,61,62,64,67,69], and post-COVID-19 [35,37,48,49,54,63]. At the country level, studies were divided into those conducted in developed countries [18,20,21,56,57,58,61,64,65,66,67,68] and those in developing countries [7,35,36,37,38,39,40,42,43,44,45,46,47,48,49,50,51,52,53,54,55,59,60,62,63,69]. In the academic discipline dimension, studies were classified into non-physical education majors [21,43,50,55,63,67,68] and physical education majors [7,35,37,39]. Regarding PA intensity, studies were grouped into LPA [43,67,69], MPA [38,43,69], MVPA [21,53,62], and VPA [43,65,69].

### 3.3. Meta-Analysis Results

**Figure 2:**
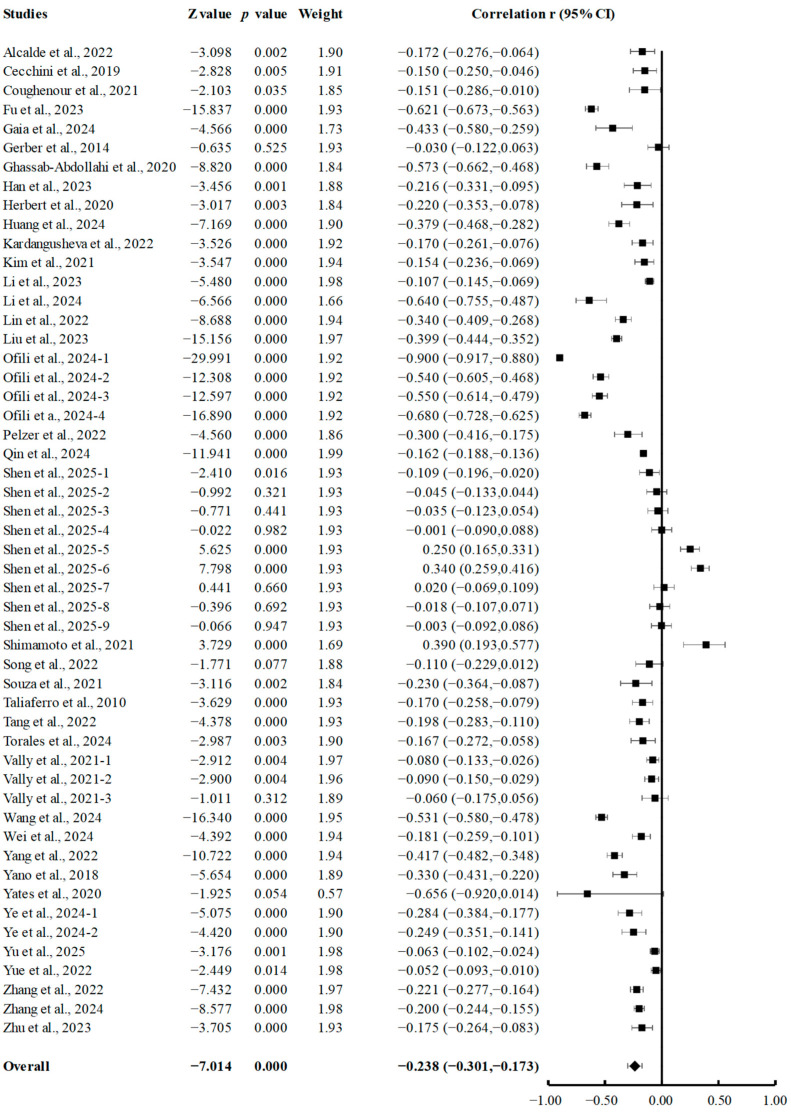
Summary of pooled correlation between PA and depression in college students [7,18,20,21,35,36,37,38,39,40,41,42,43,44,45,46,47,48,49,50,52,53,54,55,56,57,58,59,60,61,62,63,64,65,66,67,68,69].

A total of 38 studies were selected for inclusion in the meta-analysis, each investigating the correlation between PA and depression. Given the significant heterogeneity across the studies (Q = 1999.69173, *p* < 0.001; I^2^ = 97.45), a random-effects model was used. Pooled analysis demonstrated a significant negative association between PA and depression (r = −0.238; 95% CI, −0.307 to −0.173, *p* < 0.001; Figure 2).

### 3.4. Meta-Regression

To elucidate potential sources of heterogeneity, a random-effects meta-regression model was employed for further investigation. This model integrated covariates encompassing study sample size, methodological design (categorized as cross-sectional or longitudinal), and female-to-male participant ratio. The analysis identified a statistically significant association between methodological design and effect magnitude (β = −0.1679; 95% CI, −0.2835, −0.0523; *p* = 0.0044). Specifically, cross-sectional studies exhibited a more pronounced inverse association between PA and depression relative to longitudinal designs. In contrast, neither the female-to-male participant ratio (β = 0.0416; 95% CI, −0.0055, 0.0886; *p* = 0.0831) nor study sample size (β = 0.0000; 95% CI, −0.0000, 0.0001; *p* = 0.5387) demonstrated statistically significant moderation effects on the pooled effect estimate.

### 3.5. Subgroup Analysis

To further investigate the correlation between PA and depression in college students, stratified analyses were conducted based on predetermined demographic and methodological variables, aiming to examine the reliability of the combined findings and identify potential sources of variation among study cohorts.

**Figure 3:**
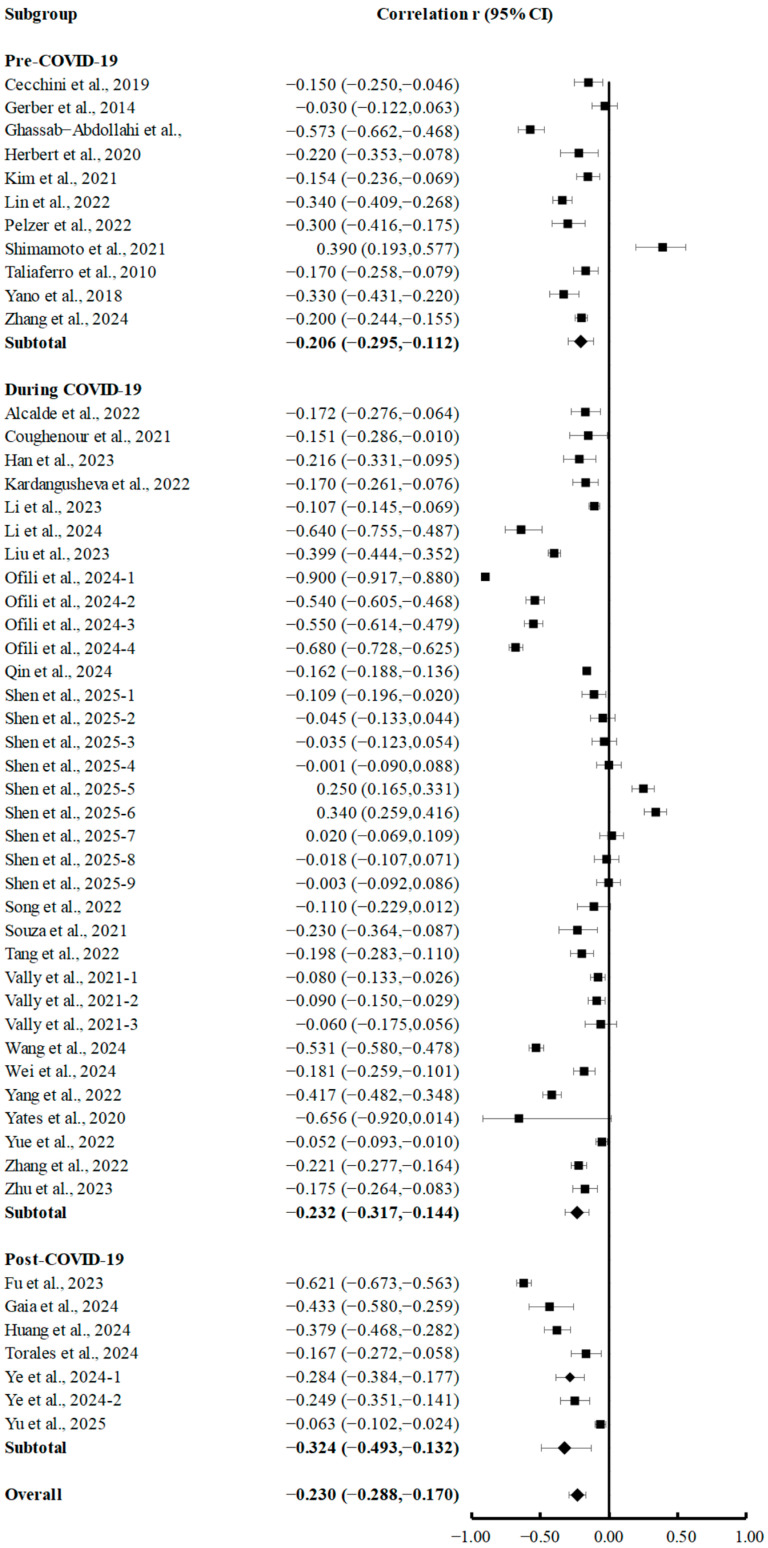
Subgroup analyses of summary correlation between PA and depression in college students before, during, and after COVID-19 [7,18,20,21,35,36,37,38,39,40,41,42,43,44,45,46,47,48,49,50,52,53,54,55,56,57,58,59,60,61,62,63,64,65,66,67,68,69].

Subgroup analyses according to the timing of data collection (pre-COVID-19, during COVID-19, and post-COVID-19) revealed significant negative correlations between PA and depression across all periods, with varying effect sizes (Figure 3). Pre-COVID-19, the combined correlation coefficient was −0.206 (95% CI, −0.295 to −0.112; *p* < 0.001), indicating a weak association (|r| = 0.10–0.30) per Cohen’s criteria. During the COVID-19 period, a similar weak negative correlation of −0.234 (95% CI, −0.321 to −0.144; *p* < 0.001) was observed. Notably, the post-COVID-19 subgroup exhibited a moderate-strength association of −0.324 (95% CI, −0.493 to −0.132; *p* < 0.001; |r| = 0.30–0.50), indicating an enhanced protective effect of PA against depression post-pandemic.

**Figure 4:**
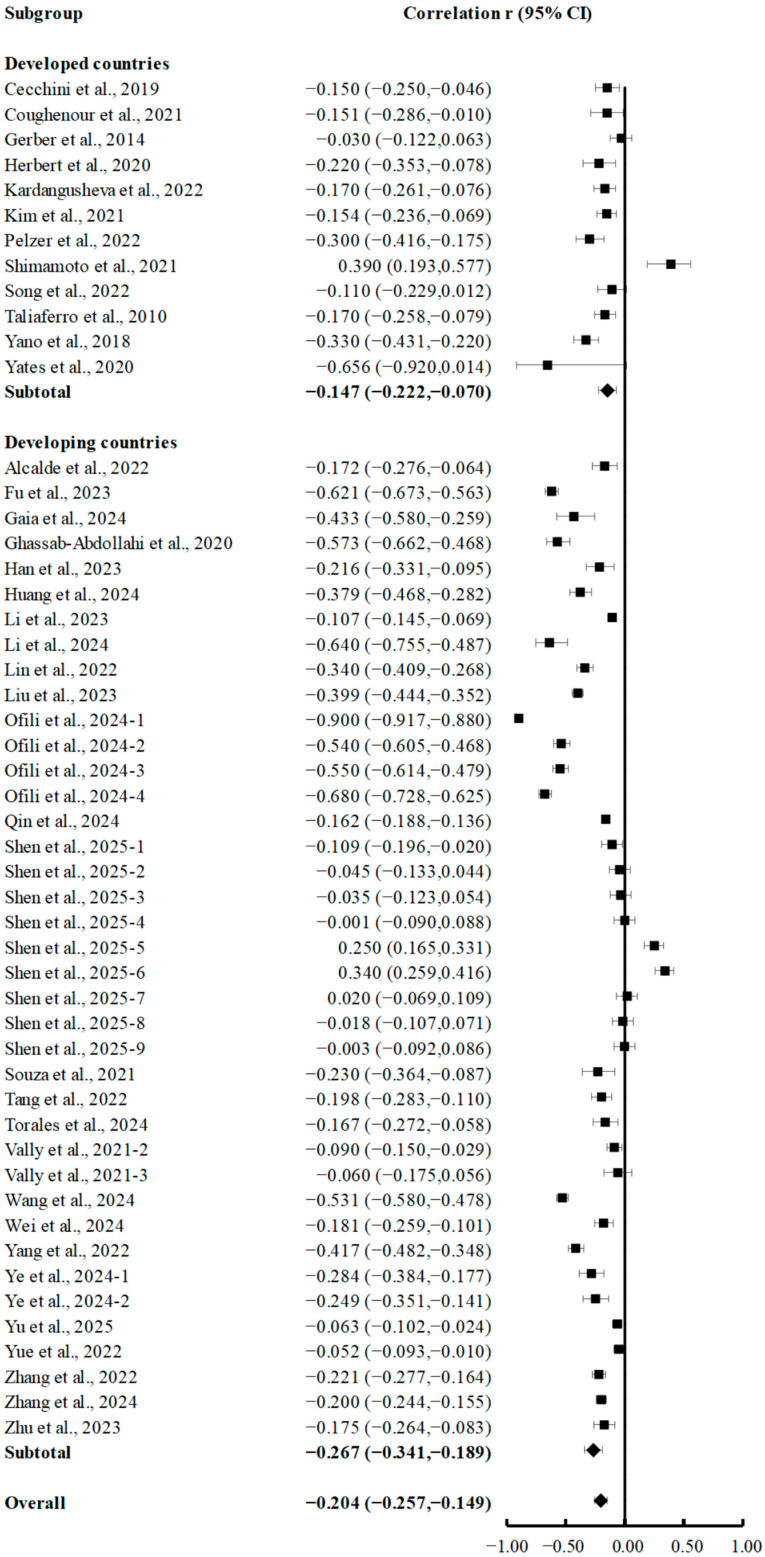
Subgroup analyses of summary correlation between PA and depression in college students from developed and developing countries [7,18,20,21,35,36,37,38,39,40,41,42,43,44,45,46,47,48,49,50,52,53,54,55,56,57,58,59,60,61,62,63,64,65,66,67,68,69].

Subgroup analyses comparing PA and depression in developed and developing countries revealed significant negative correlations in both groups. As shown in Figure 4, which presents the analysis of factors affecting the relationship between developed and developing countries, the effect sizes were −0.147 (95% CI, −0.222 to −0.070; *p* < 0.001) for developed countries and −0.202 (95% CI, −0.213 to −0.191; *p* < 0.001) for developing countries.

**Figure 5:**
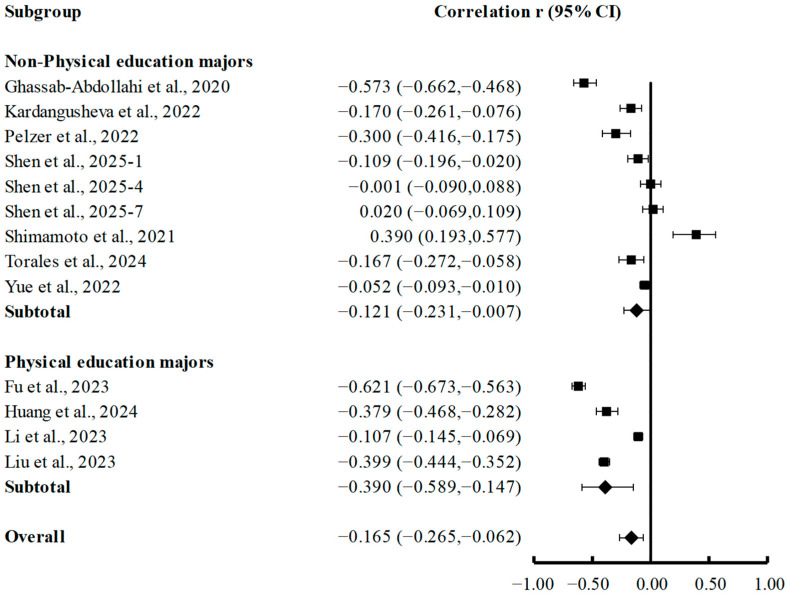
Subgroup analyses of summary correlation between PA and depression in college students majoring in physical education and non-physical education [7,21,35,37,39,43,50,55,63,67,68].

Subgroup analyses by academic discipline (non-physical education majors and physical education majors) revealed notable differences in the correlation between PA and depression (Figure 5). Among non-physical education majors, a modest yet statistically significant negative correlation was found (r = −0.121; 95% CI, −0.231 to −0.007; *p* = 0.037), falling within the small effect size range (|r| = 0.10–0.30). In contrast, physical education majors exhibited a significantly stronger negative correlation (r = −0.390; 95% CI, −0.589 to −0.147; *p* < 0.001), indicating a medium effect size (|r| = 0.30–0.50).

**Figure 6:**
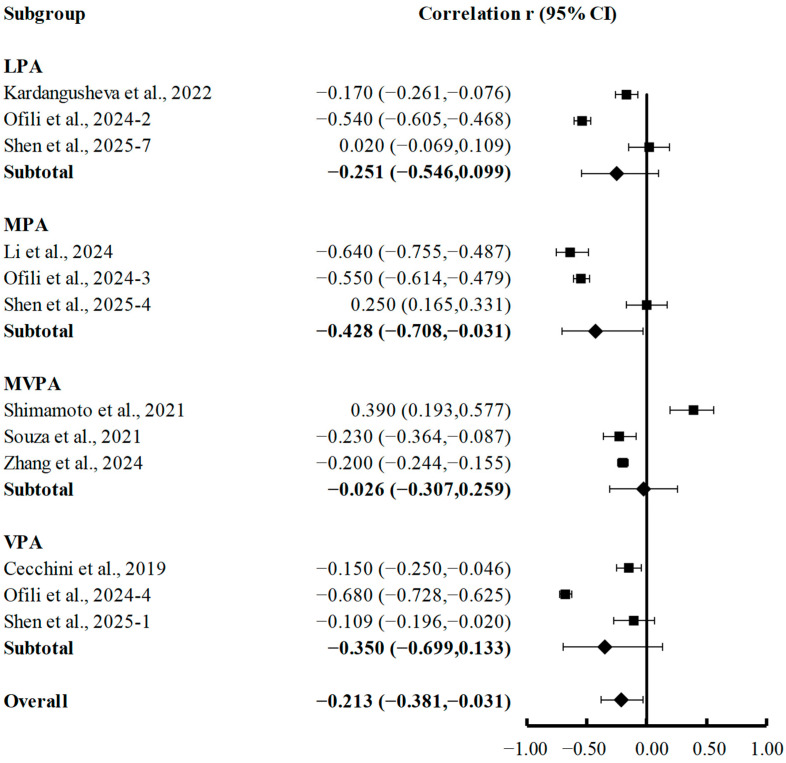
Subgroup analyses of summary correlation between LPA, MPA, MVPA, VPA, and depression in college students [21,38,43,53,62,65,67,69].

Subgroup analyses based on different PA intensities revealed distinct associations with depression in college students. As presented in Figure 6, LPA showed no significant correlation with depression (r = −0.251; 95% CI, −0.546 to 0.099; *p* = 0.157). MVPA also demonstrated a non-significant weak negative association (r = −0.026; 95% CI, −0.307 to 0.259; *p* = 0.859). However, MPA exhibited a statistically significant moderate negative correlation (r = −0.428; 95% CI, −0.708 to −0.031; *p* = 0.035), with its 95% CI entirely having fallen below the null line, suggesting robust protective effects. VPA showed a clinically meaningful effect size (r = −0.350), but its association was not statistically significant (95% CI, −0.699 to 0.133; *p* = 0.151) due to study variability. The stronger effect of MPA compared to other intensities (approximately 1.6 times stronger than VPA) implied a potential optimal activity intensity for mental health benefits, with insufficient (LPA) and excessive (VPA) activity levels appearing less effective in alleviating depression.

### 3.6. Risk of Bias

The methodological quality of the included studies was evaluated using the JBI Critical Appraisal Checklist. As presented in Table S4, all studies scored above 14 out of 20 points, indicating a low risk of bias as per JBI criteria. The high methodological quality of the included studies enhances the robustness of the meta-analytic synthesis.

### 3.7. Publication Bias

As presented in Figure S1, the funnel plot utilized to evaluate publication bias within the meta-analysis illustrated the relationship between Fisher’s Z values and their standard errors. The plot revealed a concentration of effect estimates primarily near the apex of the funnel, with visual inspection indicating no overt asymmetry. This pattern suggested limited evidence of substantial publication bias.

### 3.8. Sensitivity Analysis

In the sequential analysis where studies were systematically excluded, no outliers were detected. The correlation coefficient ranged from r = −0.248 to −0.213 for each study removal, indicating that no individual study had a substantial impact on the correlation coefficient or the meta-analysis results. Following sensitivity analysis, the correlation between PA and depression remained statistically significant (summary r = −0.238, 95% CI, −0.301 to −0.173, Figure S2). Consequently, the findings can be deemed as robust and trustworthy. Additionally, the Egger’s tests indicated non-significant publication bias (*p* = 0.11).

## 4. Discussion

The present study established a moderate negative correlation between PA and depression in college students, aligning with previous research [70,71]. PA has been recognized as an effective treatment for depression [72]. Current evidence suggests that the relationship between PA and depression is mediated by multiple psychosocial and neurobiological mechanisms [73]. PA enhances self-esteem, reduces loneliness, alleviates perceived stress, anxiety, and improves self-efficacy through striatal dopamine modulation [18,35,41,43,44,45,46,66]. Specifically, PA elevates self-esteem in college students, thereby lowering depression risk [18,35,45,46]. Team sports participation fosters social connections, reduces loneliness, and subsequently decreases depression rates [18]. PA also mitigates depression by reducing stress and enhancing self-efficacy [43,44]. However, a previous study identified a positive correlation between high PA levels and depression [21]. This discrepancy may be attributed to the high-pressure academic environment in Japanese universities, where students face prolonged stress from university entrance examinations. Upon entering university, students may seek psychological balance through social activities but risk overexertion. Japanese college students often experience academic, work-related, and daily life stressors, leading to a “fatigue–depression” cycle accompanied by insomnia. Additionally, athletes or student-athletes, who constitute a significant portion of high PA participants, require adequate sleep for recovery. Sleep deprivation may exacerbate mental health issues [74].

Another study reported a positive correlation between light-to-moderate PA and depression in college students [43]. This relationship may stem from the limited social aspects of light-to-moderate PA, which often focuses on training or competition rather than social interaction. Furthermore, a prior study found no significant association between aerobic exercise and depression in college students [20]. This lack of association may be due to students perceiving routine activities like biking to campus as aerobic PA. These low-intensity, unstructured activities may not sufficiently activate the neurobiological processes responsible for mood enhancement. While the main results indicated a negative link between PA and depression, various mediating factors influence this relationship. Differences in study outcomes may arise from confounding variables.

MPA emerged as the most significant intensity threshold, demonstrating a stronger negative correlation with depression in college students compared to LPA, MVPA, and VPA. Specifically, MPA exhibited a stronger relationship than LPA, MVPA, and VPA. These results underscore the importance of modulating activity intensity for exercise interventions targeting student mental health. Notably, only MPA had a 95% CI entirely below the null line, while other PA levels crossed the null line. This finding suggested that MPA is optimal for improving emotional regulation in college students [70,75]. Paolucci et al. [76] proposed that MPA enhances mental health by lowering tumor necrosis factor-alpha (TNF-α) levels. Insufficient intensity may fail to alleviate negative emotions [43,67,69], whereas excessive intensity could lead to adverse effects such as sleep disturbances or fatigue [77]. These results are consistent with previous meta-analyses reporting a negative association between MPA and depression across diverse populations [78]. Additionally, Laske et al. [79] investigated the mechanisms through which MPA mitigates depression from physiological, neurobiological, and psychosocial perspectives. This exercise type enhances neuroplasticity and neurotrophic factors, lowers inflammatory markers, improves oxidative stress regulation, and fosters psychosocial health.

By maintaining a balance between effective stimulation and avoiding excessive stress responses, MPA maximizes inflammation reduction, prevents cortisol spikes, and significantly increases serum brain-derived neurotrophic factor (BDNF) levels, thereby improving neuronal survival and function. Beyond mental well-being, MPA also enhances cardiovascular function, metabolic markers, and sleep quality. These physiological benefits indirectly mitigate depression through mind–body interaction [80]. Consequently, MPA demonstrated a significantly stronger negative correlation with depression in college students compared to other PA intensities.

Post-COVID-19, the correlation between PA and depression was stronger than pre-COVID-19 and during COVID-19. These temporally stratified results suggest that COVID-19 may act as an effect modifier in the relationship between PA and depression in college students. This heightened correlation may be attributed to the pandemic’s significant global socioeconomic and public health impacts, leading to increased uncertainty about the future and subsequently exacerbating anxiety and depression. A meta-analysis projected an additional 76.2 million anxiety disorder cases globally (an increase of 25.6% [23.2 to 28.0]) [81]. During the pandemic, college students experienced reduced PA and poor dietary choices due to campus lockdowns, resulting in diminished physical fitness and severe mental health repercussions [82]. Post-pandemic, heightened awareness of PA in college students correlated with increased PA levels and improved outcomes. Despite elevated baseline depression post-pandemic, PA represents a cost-effective approach for treating and preventing depression, offering significant advantages in the post-pandemic economic downturn [83]. Hence, COVID-19 plays a pivotal role in shaping the association between PA and depression, with PA exhibiting a heightened impact on mitigating depression post-pandemic.

The correlation between PA and depression was similar in developed countries and developing countries. According to Cohen’s criteria for correlation strength, these findings suggested that PA offers similarly modest protection against depression in college students regardless of national economic development status, with no substantial difference detected between developed and developing countries. This uniformity in effect sizes implies that the protective link between PA and depression may transcend socioeconomic disparities. The comparable 95% CIs indicate similar precision, likely due to sufficient sample sizes and measurement consistency. Despite cultural and healthcare system differences between these country groups, the results suggested that the biological mechanisms connecting PA to mental health outcomes operate independently of national development levels.

This consistency across diverse contexts strengthens the rationale for global mental health interventions promoting PA. The comparable effect sizes may imply that national-level socioeconomic factors, while potentially influencing baseline depression rates, do not significantly modify the fundamental relationship between PA and depression in college students. Developing countries may still possess advantages, possibly due to significant economic disparities compared to developed countries. College students in low- and middle-income countries reside in resource-limited environments, rendering them highly susceptible to mental health challenges [84]. Economic development can exacerbate depression and anxiety in college students [85]. Additionally, Sacks et al. [86] suggested a clear correlation between economic growth and well-being. Consequently, college students in developing countries may derive more pronounced benefits from PA in alleviating depression.

Physical education majors demonstrated a more pronounced effect in mitigating depression compared to non-physical education majors. These results corroborate prior findings suggesting that physical education students derive greater mental health benefits from PA compared to their non-physical education peers. The current meta-analysis highlighted approximately a threefold increase in protective effects against depression in students specializing in physical education disciplines. A previous meta-analysis indicated that physical education can enhance positive emotions and mental well-being in students [87]. Furthermore, Fu et al. [35] proposed that sports majors often experience skill validation through training and competitions, leading to enhanced self-efficacy. Huang et al. [37] argued that continuous intense training helps sports majors build increased psychological resilience in areas such as goal-setting, teamwork, and managing setbacks. Consequently, physical education students are better equipped than their non-physical education counterparts to utilize PA for preventing depression.

Sleep characteristics, dietary patterns, and socioeconomic status represent potential confounding variables influencing depression in college students. Existing evidence indicates that suboptimal sleep quality is negatively correlated with mental health outcomes [88]. Dietary habits differ across cultural contexts, and these variations are linked to varying degrees of depression risk [89]. Lower socioeconomic status consistently demonstrates an association with increased depression prevalence [90]. Consequently, these covariates may confound the observed relationship between physical activity and depression in collegiate populations.

Despite the strengths of this analysis, several limitations warrant acknowledgement. First, the included studies displayed significant heterogeneity. Although four distinct subgroup analyses were conducted based on pre/post-pandemic periods, countries, disciplines, and PA intensities, no factors were identified to explain this heterogeneity. Second, the substantial variation in the information provided by the included studies precluded a quantitative evaluation of their impact on heterogeneity. Most included studies employed a cross-sectional design, introducing confounding variables that may compromise result objectivity and potentially inflate the association between PA and depression. The observed heterogeneity across study outcomes can be systematically interpreted through the PECO framework. Within the Population domain, the inclusion of participants from diverse geographical regions with minimal health status restrictions introduced substantial demographic and cultural heterogeneity. For Exposure, significant variations in PA intervention types (e.g., aerobic, resistance) and intensity protocols (e.g., LPA, MVPA) across studies contributed to methodological inconsistency. The Comparison component exhibited variability due to inconsistent eligibility criteria and disparate definitions of control/reference groups among included studies. Regarding Outcome assessment, non-standardized measurement tools (e.g., CES-D, PHQ-9 for depression; IPAQ, accelerometry for PA) reduced data comparability. Additional sources of bias include potential language publication bias from exclusive inclusion of English literature and reduced statistical power from limited sample sizes. These factors—heterogeneous population characteristics, divergent exposure parameters, inconsistent comparison methodologies, and non-uniform outcome measures—collectively account for the significant heterogeneity observed, potentially influencing the synthesized findings. Notably, the cross-sectional design of most included studies precludes causal inference between PA and depression. Future research should prioritize longitudinal cohorts or RCTs to establish causal relationships. Nevertheless, engagement in MPA remains empirically supported as a preventive behavioral intervention against depressive symptomatology.

## 5. Conclusions

This systematic review and meta-analysis suggests that PA is significantly and negatively associated with depression and plays a crucial role in alleviating depression in college students. Various influences, including the pandemic, national development level, student major, and PA intensity, moderate this relationship. Post-pandemic, developing countries, physical education majors, and moderate PA intensity emerged as optimal factors for enhancing the depression-alleviating effects of PA.

## Data Availability

The original contributions presented in the study are included in the article/Supplementary Materials, further inquiries can be directed to the corresponding author.

## Supplementary Materials

The following supporting information can be downloaded at: https://www.mdpi.com/article/10.3390/brainsci15080875/s1, Figure S1: Funnel plot; Figure S2: Sensitivity analysis results; Table S1: Search strategies; Table S2: Excluded studies list; Table S3: Characteristics of studies included in this meta-analysis; Table S4: Details of the scoring criteria in the JBI appraisal checklist.

## Abbreviations

The following abbreviations are used in this manuscript:

PA	physical activity
CBT	cognitive behavioral therapy
OCD	obsessive–compulsive disorder
PTSD	post-traumatic stress disorder
COVID-19	coronavirus disease 2019
SDS	self-rating depression scale
BDI	Beck depression inventory
JBI	Joanna Briggs Institute
LPA	low physical activity
MPA	moderate physical activity
MVPA	moderate-to-vigorous physical activity
VPA	vigorous physical activity
BDNF	brain-derived neurotrophic factor
