# Supplementary material for: The Relationship Between Physical Activity and Depression in College Students: A Systematic Review and Meta-Analysis
Source: Brain Sci. 2025 Aug 18;15(8):875. doi: 10.3390/brainsci15080875 (PMC12385083; doi:10.3390/brainsci15080875)
Supplement: Supplementary file 1 [file brainsci-15-00875-s001.zip › brainsci-3803978-supplementary.pdf]

Supplemental material

The relationship between physical activity and depression in college students: a systematic review and meta-analysis

Figure S1. Funnel plot..2

Figure S2. Sensitivity analysis results 3

Table S1. Search strategies 4

Table S2. Excluded studies list 9

Table S3. Characteristics of studies included in this meta-analysis............................18

Table S4. Details of the scoring criteria in the JBI appraisal checklist........................26

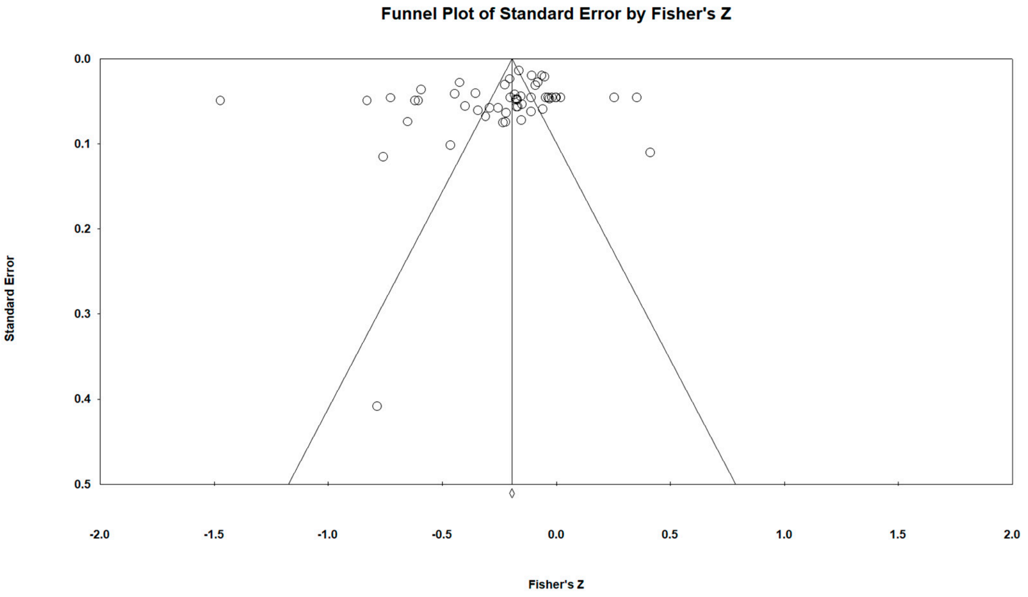

Figure S1. Funnel plot.

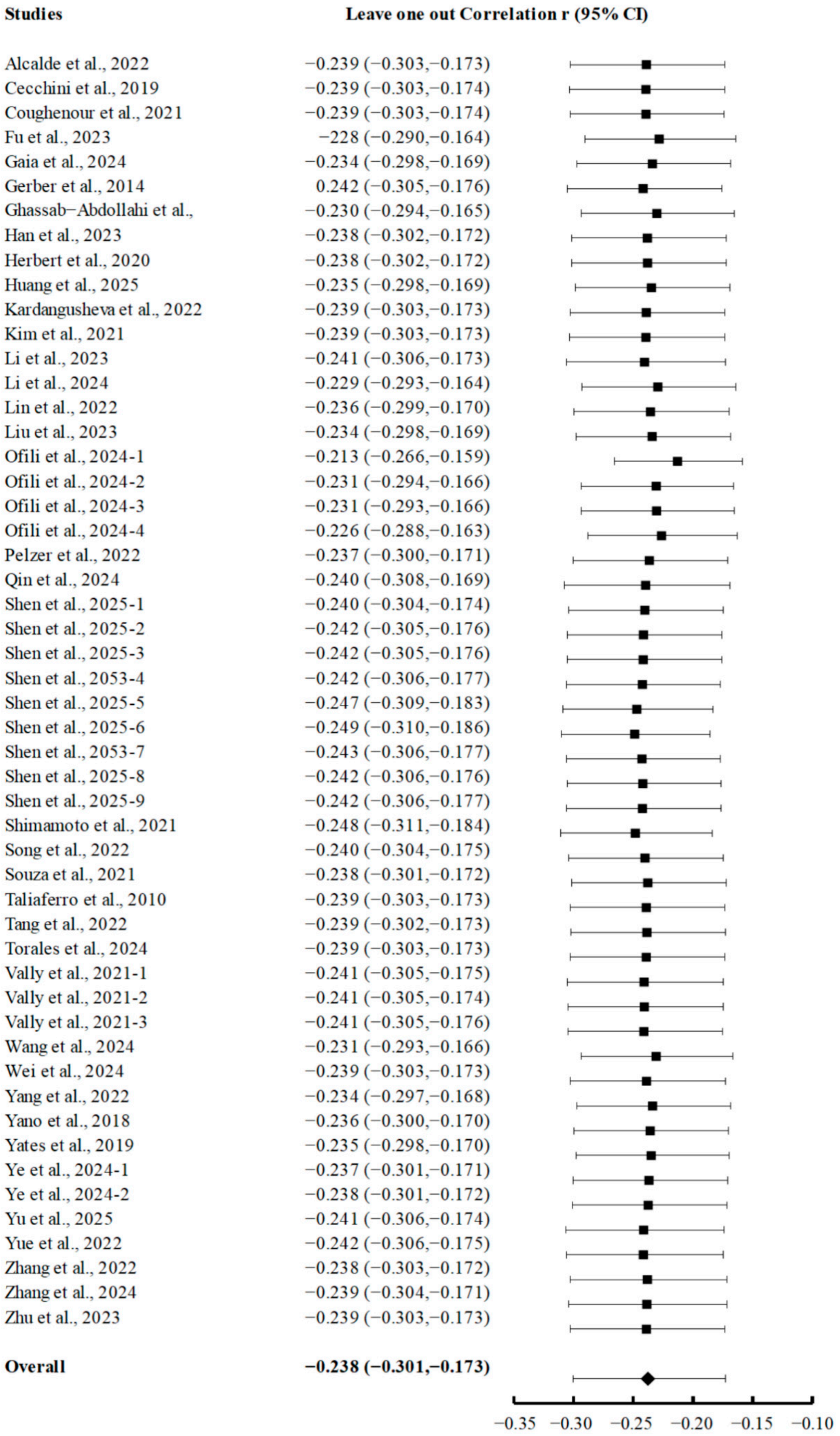

Figure S2. Sensitivity analysis [7,18,20,21,35-50,52-69].

Table S1. Search strategies.

**Web of Science**
#1<br>(TS=("Exercise") OR TS=("Exercises") OR TS=("Exercise, Physical") OR TS=("Exercises, Physical") OR TS=("Physical Exercise") OR TS=("Physical Exercises") OR TS=("Exercise, Aerobic") OR TS=("Aerobic Exercise") OR TS=("Aerobic Exercises") OR TS=("Exercises, Aerobic") OR TS=("Exercise, Isometric") OR TS=("Exercises, Isometric") OR TS=("Isometric Exercises") OR TS=("Isometric Exercise") OR TS=("Acute Exercise") OR TS=("Acute Exercises") OR TS=("Exercise, Acute") OR TS=("Exercises, Acute") OR TS=("Exercise Training") OR TS=("Exercise Trainings") OR TS=("Training, Exercise") OR TS=("Trainings, Exercise") OR TS=("Physical Activity") OR TS=("Activities, Physical") OR TS=("Activity, Physical") OR TS=("Physical Activities"))<br>#2<br>(TS=("Depression") OR TS=("Depressive Symptoms") OR TS=("Depressive Symptom") OR TS=("Symptom, Depressive") OR TS=("Emotional Depression") OR TS=("Depression, Emotional"))<br>#3<br>(TS=("Students, Undergraduate") OR TS=("Students, College") OR TS=("Students, Graduate") OR TS=("College Graduates") OR TS=("College Student*") OR TS=("College*") OR TS=("Universit*") OR TS=("University Student*") OR TS=("University Man") OR TS=("Higher Education") OR TS=("Postsecondary") OR TS=("Post Secondary") OR TS=("Graduates") OR TS=("Undergraduate*") OR TS=("Freshman") OR TS=("Freshmen") OR TS=("Sophomore*") OR TS=("Junior*") OR TS=("Senior*") OR TS=("Graduate Student") OR TS=("Graduate Education") OR TS=("Undergraduate Student'") OR TS=("Education, Graduate") OR TS=("Educations, Graduate") OR TS=("Students, Nurse Midwifery") OR TS=("Students, Nursing, Associate") OR TS=("Students, Nursing, Doctoral") OR TS=("Students, Nursing, Male") OR TS=("Students, Nursing, Baccalaureate") OR TS=("Students, Nursing, Diploma Programs") OR TS=("Students, Nursing, Masters") OR TS=("Students, Nursing") OR TS=("Students, Medical") OR TS=("Students, Nursing, Practical") OR TS=("Students, Respiratory Therapy") OR TS=("Students, Audiology") OR TS=("Students, Dental") OR TS=("Students, Chiropractic") OR TS=("Students, Dental Hygiene") OR TS=("Community Colleges") OR TS=("Academician") OR TS=("College-Goer*"))<br>#1 and #2 and #3
**PubMed**
#1<br>("Exercise"[Title/Abstract] or "Exercises"[Title/Abstract] or "Exercise, Physical"[Title/Abstract] or "Exercises, Physical"[Title/Abstract] or "Physical Exercise"[Title/Abstract] or "Physical Exercises"[Title/Abstract] or "Exercise, Aerobic"[Title/Abstract] or "Aerobic Exercise"[Title/Abstract] or "Aerobic

Exercises"[Title/Abstract] or "Exercises, Aerobic"[Title/Abstract] or "Exercise, Isometric"[Title/Abstract] or "Exercises, Isometric"[Title/Abstract] or "Isometric Exercises"[Title/Abstract] or "Isometric Exercise"[Title/Abstract] or "Acute Exercise"[Title/Abstract] or "Acute Exercises"[Title/Abstract] or "Exercise, Acute"[Title/Abstract] or "Exercises, Acute"[Title/Abstract] or "Exercise Training"[Title/Abstract] or "Exercise Trainings"[Title/Abstract] or "Training, Exercise"[Title/Abstract] or "Trainings, Exercise"[Title/Abstract] or "Physical Activity"[Title/Abstract] or "Activities, Physical"[Title/Abstract] or "Activity, Physical"[Title/Abstract] or "Physical Activities"[Title/Abstract])<br>#2<br>("Depression"[Title/Abstract] or "Depressive Symptoms"[Title/Abstract] or "Depressive Symptom"[Title/Abstract] or "Symptom, Depressive"[Title/Abstract] or "Emotional Depression"[Title/Abstract] or "Depression, Emotional"[Title/Abstract])<br>#3<br>("Students, Undergraduate"[Title/Abstract] or "Students, College"[Title/Abstract] or "Students, Graduate"[Title/Abstract] or "College Graduates"[Title/Abstract] or "College Student*"[Title/Abstract] or "College*"[Title/Abstract] or "Universit*"[Title/Abstract] or "University Student*"[Title/Abstract] or "University Man"[Title/Abstract] or "Higher Education"[Title/Abstract] or "Postsecondary"[Title/Abstract] or "Post Secondary"[Title/Abstract] or "Graduates"[Title/Abstract] or "Undergraduate*"[Title/Abstract] or "Freshman"[Title/Abstract] or "Freshmen"[Title/Abstract] or "Sophomore*"[Title/Abstract] or "Junior*"[Title/Abstract] or "Senior*"[Title/Abstract] or "Graduate Student"[Title/Abstract] or "Graduate Education"[Title/Abstract] or "Undergraduate Student' "[Title/Abstract] or "Education, Graduate"[Title/Abstract] or "Educations, Graduate"[Title/Abstract] or "Students, Nurse Midwifery"[Title/Abstract] or "Students, Nursing, Associate"[Title/Abstract] or "Students, Nursing, Doctoral"[Title/Abstract] or "Students, Nursing, Male"[Title/Abstract] or "Students, Nursing, Baccalaureate"[Title/Abstract] or "Students, Nursing, Diploma Programs"[Title/Abstract] or "Students, Nursing, Masters"[Title/Abstract] or "Students, Nursing"[Title/Abstract] or "Students, Medical"[Title/Abstract] or "Students, Nursing, Practical"[Title/Abstract] or "Students, Respiratory Therapy"[Title/Abstract] or "Students, Audiology"[Title/Abstract] or "Students, Dental"[Title/Abstract] or "Students, Chiropractic"[Title/Abstract] or "Students, Dental Hygiene"[Title/Abstract] or "Community Colleges"[Title/Abstract] or "Academician"[Title/Abstract] or "College-Goer*"[Title/Abstract])<br>#1 and #2 and #3
**Cochrane**
#1<br>("Exercise"):ti,ab,kw or ("Exercises"):ti,ab,kw or ("Exercise, Physical"):ti,ab,kw or ("Exercises, Physical"):ti,ab,kw or ("Physical Exercise"):ti,ab,kw or ("Physical Exercises"):ti,ab,kw or ("Exercise, Aerobic"):ti,ab,kw or ("Aerobic Exercise"):ti,ab,kw or ("Aerobic Exercises"):ti,ab,kw or ("Exercises,

Aerobic"):ti,ab,kw or ("Exercise, Isometric"):ti,ab,kw or ("Exercises, Isometric"):ti,ab,kw or ("Isometric Exercises"):ti,ab,kw or ("Isometric Exercise"):ti,ab,kw or ("Acute Exercise"):ti,ab,kw or ("Acute Exercises"):ti,ab,kw or ("Exercise, Acute"):ti,ab,kw or ("Exercises, Acute"):ti,ab,kw or ("Exercise Training"):ti,ab,kw or ("Exercise Trainings"):ti,ab,kw or ("Training, Exercise"):ti,ab,kw or ("Trainings, Exercise"):ti,ab,kw or ("Physical Activity"):ti,ab,kw or ("Activities, Physical"):ti,ab,kw or ("Activity, Physical"):ti,ab,kw or ("Physical Activities"):ti,ab,kw<br>#2<br>("Depression"):ti,ab,kw or ("Depressive Symptoms"):ti,ab,kw or ("Depressive Symptom"):ti,ab,kw or ("Symptom, Depressive"):ti,ab,kw or ("Emotional Depression"):ti,ab,kw or ("Depression, Emotional"):ti,ab,kw<br>#3<br>("Students, Undergraduate"):ti,ab,kw or ("Students, College"):ti,ab,kw or ("Students, Graduate"):ti,ab,kw or ("College Graduates"):ti,ab,kw or (College NEXT Student*):ti,ab,kw or (College*):ti,ab,kw or (Universit*):ti,ab,kw or (University NEXT Student*):ti,ab,kw or ("University Man"):ti,ab,kw or ("Higher Education"):ti,ab,kw or ("Postsecondary"):ti,ab,kw or ("Post Secondary"):ti,ab,kw or ("Graduates"):ti,ab,kw or (N Undergraduate*):ti,ab,kw or (Freshman):ti,ab,kw or ("Freshmen"):ti,ab,kw or (Sophomore*):ti,ab,kw or (Junior*):ti,ab,kw or (Senior*):ti,ab,kw or ("Graduate Student"):ti,ab,kw or ("Graduate Education"):ti,ab,kw or ("Undergraduate Student'"):ti,ab,kw or ("Education, Graduate"):ti,ab,kw or ("Educations, Graduate"):ti,ab,kw or ("Students, Nurse Midwifery"):ti,ab,kw or ("Students, Nursing, Associate"):ti,ab,kw or ("Students, Nursing, Doctoral"):ti,ab,kw or ("Students, Nursing, Male"):ti,ab,kw or ("Students, Nursing, Baccalaureate"):ti,ab,kw or ("Students, Nursing, Diploma Programs"):ti,ab,kw or ("Students, Nursing, Masters"):ti,ab,kw or ("Students, Nursing"):ti,ab,kw or ("Students, Medical"):ti,ab,kw or ("Students, Nursing, Practical"):ti,ab,kw or ("Students, Respiratory Therapy"):ti,ab,kw or ("Students, Audiology"):ti,ab,kw or ("Students, Dental"):ti,ab,kw or ("Students, Chiropractic"):ti,ab,kw or ("Students, Dental Hygiene"):ti,ab,kw or ("Community Colleges"):ti,ab,kw or ("Academician"):ti,ab,kw or (College-Goer*)<br>#1 and #2 and #3
**Embase**
#1<br>'Exercise':ab,ti or 'Exercises':ab,ti or 'Exercise, Physical':ab,ti or 'Exercises, Physical':ab,ti or 'Physical Exercise':ab,ti or 'Physical Exercises':ab,ti or 'Exercise, Aerobic':ab,ti or 'Aerobic Exercise':ab,ti or 'Aerobic Exercises':ab,ti or 'Exercises, Aerobic':ab,ti or 'Exercise, Isometric':ab,ti or 'Exercises, Isometric':ab,ti or 'Isometric Exercises':ab,ti or 'Isometric Exercise':ab,ti or 'Acute Exercise':ab,ti or 'Acute Exercises':ab,ti or 'Exercise, Acute':ab,ti or 'Exercises, Acute':ab,ti or 'Exercise Training':ab,ti or 'Exercise Trainings':ab,ti or 'Training, Exercise':ab,ti or 'Trainings, Exercise':ab,ti or 'Physical Activity':ab,ti or 'Activities, Physical':ab,ti or 'Activity, Physical':ab,ti or 'Physical Activities':ab,ti

#2<br>'Depression':ab,ti or 'Depressive Symptoms':ab,ti or 'Depressive Symptom':ab,ti or 'Symptom, Depressive':ab,ti or 'Emotional Depression':ab,ti or 'Depression, Emotional':ab,ti<br>#3<br>'Students, Undergraduate':ab,ti or 'Students, College':ab,ti or 'Students, Graduate':ab,ti or 'College Graduates':ab,ti or 'College Student*':ab,ti or 'College*':ab,ti or 'Universit*':ab,ti or 'University Student*':ab,ti or 'University Man':ab,ti or 'Higher Education':ab,ti or 'Postsecondary':ab,ti or 'Post Secondary':ab,ti or 'Graduates':ab,ti or 'Undergraduate*':ab,ti or 'Freshman':ab,ti or 'Freshmen':ab,ti or 'Sophomore*':ab,ti or 'Junior*':ab,ti or 'Senior*':ab,ti or 'Graduate Student':ab,ti or 'Graduate Education':ab,ti or 'Undergraduate Student':ab,ti or 'Education, Graduate':ab,ti or 'Educations, Graduate':ab,ti or 'Students, Nurse Midwifery':ab,ti or 'Students, Nursing, Associate':ab,ti or 'Students, Nursing, Doctoral':ab,ti or 'Students, Nursing, Male':ab,ti or 'Students, Nursing, Baccalaureate':ab,ti or 'Students, Nursing, Diploma Programs':ab,ti or 'Students, Nursing, Masters':ab,ti or 'Students, Nursing':ab,ti or 'Students, Medical':ab,ti or 'Students, Nursing, Practical':ab,ti or 'Students, Respiratory Therapy':ab,ti or 'Students, Audiology':ab,ti or 'Students, Dental':ab,ti or 'Students, Chiropractic':ab,ti or 'Students, Dental Hygiene':ab,ti or 'Community Colleges':ab,ti or 'Academician':ab,ti or 'College - Goer*':ab,ti<br>#1 and #2 and #3
**Scopus**
(TITLE-ABS-KEY("Exercise") OR TITLE-ABS-KEY("Exercises") OR TITLE-ABS-KEY("Exercise, Physical") OR TITLE-ABS-KEY("Exercises, Physical") OR TITLE-ABS-KEY("Physical Exercise") OR TITLE-ABS-KEY("Physical Exercises") OR TITLE-ABS-KEY("Exercise, Aerobic") OR TITLE-ABS-KEY("Aerobic Exercise") OR TITLE-ABS-KEY("Aerobic Exercises") OR TITLE-ABS-KEY("Exercises, Aerobic") OR TITLE-ABS-KEY("Exercise, Isometric") OR TITLE-ABS-KEY("Exercises, Isometric") OR TITLE-ABS-KEY("Isometric Exercises") OR TITLE-ABS-KEY("Isometric Exercise") OR TITLE-ABS-KEY("Acute Exercise") OR TITLE-ABS-KEY("Acute Exercises") OR TITLE-ABS-KEY("Exercise, Acute") OR TITLE-ABS-KEY("Exercises, Acute") OR TITLE-ABS-KEY("Exercise Training") OR TITLE-ABS-KEY("Exercise Trainings") OR TITLE-ABS-KEY("Training, Exercise") OR TITLE-ABS-KEY("Trainings, Exercise") OR TITLE-ABS-KEY("Physical Activity") OR TITLE-ABS-KEY("Activities, Physical") OR TITLE-ABS-KEY("Activity, Physical") OR TITLE-ABS-KEY("Physical Activities")) AND (TITLE-ABS-KEY("Depression") OR TITLE-ABS-KEY("Depressive Symptoms") OR TITLE-ABS-KEY("Depressive Symptom") OR TITLE-ABS-KEY("Symptom, Depressive") OR TITLE-ABS-KEY("Emotional Depression") OR TITLE-ABS-KEY("Depression, Emotional")) AND (TITLE-ABS-KEY("Students, Undergraduate") OR TITLE-ABS-KEY("Students, College") OR TITLE-ABS-KEY("Students, Graduate") OR TITLE-ABS-KEY("College Graduates") OR

TITLE-ABS-KEY("College Student*") OR TITLE-ABS-KEY("College*") OR TITLE-ABS-KEY("Universit*") OR TITLE-ABS-KEY("University Student*") OR TITLE-ABS-KEY("University Man") OR TITLE-ABS-KEY("Higher Education") OR TITLE-ABS-KEY("Postsecondary") OR TITLE-ABS-KEY("Post Secondary") OR TITLE-ABS-KEY("Graduates") OR TITLE-ABS-KEY("Undergraduate*") OR TITLE-ABS-KEY("Freshman") OR TITLE-ABS-KEY("Freshmen") OR TITLE-ABS-KEY("Sophomore*") OR TITLE-ABS-KEY("Junior*") OR TITLE-ABS-KEY("Senior*") OR TITLE-ABS-KEY("Graduate Student") OR TITLE-ABS-KEY("Graduate Education") OR TITLE-ABS-KEY("Undergraduate Student") OR TITLE-ABS-KEY("Education, Graduate") OR TITLE-ABS-KEY("Educations, Graduate") OR TITLE-ABS-KEY("Students, Nurse Midwifery") OR TITLE-ABS-KEY("Students, Nursing, Associate") OR TITLE-ABS-KEY("Students, Nursing, Doctoral") OR TITLE-ABS-KEY("Students, Nursing, Male") OR TITLE-ABS-KEY("Students, Nursing, Baccalaureate") OR TITLE-ABS-KEY("Students, Nursing, Diploma Programs") OR TITLE-ABS-KEY("Students, Nursing, Masters") OR TITLE-ABS-KEY("Students, Nursing") OR TITLE-ABS-KEY("Students, Medical") OR TITLE-ABS-KEY("Students, Nursing, Practical") OR TITLE-ABS-KEY("Students, Respiratory Therapy") OR TITLE-ABS-KEY("Students, Audiology") OR TITLE-ABS-KEY("Students, Dental") OR TITLE-ABS-KEY("Students, Chiropractic") OR TITLE-ABS-KEY("Students, Dental Hygiene") OR TITLE-ABS-KEY("Community Colleges") OR TITLE-ABS-KEY("Academician") OR TITLE-ABS-KEY("College-Goer*"))
#1 and #2 and #3

Table S2. Excluded studies list.

**Reasons**	**Title**

Association between physical activity and depression not investigated (n = 79)	1. Associations between takeaway food consumption, physical activity levels and their joint effect with comorbid depression and anxiety symptoms among Chinese university students<br><br>2. The mediating roles of sleep quality and sedentary behavior between physical fitness and depression among female college freshmen<br><br>3. Longitudinal effects of motivation and physical activity on depressive symptoms among college students<br><br>4. Research on the intervention effect of five-element music combined with eight-section brocade on depression among medical students in higher vocational colleges<br><br>5. Physical literacy and health of Chinese medical students: the chain mediating role of physical activity and subjective well-being<br><br>6. Collective rehabilitation training conductive to improve psychotherapy of college students with anxiety disorder<br><br>7. Psychophysiological effects of rhythmic music combined with aerobic exercise in college students with minimal depressive symptoms<br><br>8. Women's college physical activity and self-reports of physician-diagnosed depression and of current symptoms of psychiatric distress<br><br>9. A comparison of the effects of remote coaching hiit training and combined exercise training on the physical and mental health of university students<br><br>10. Alterations in selected measures of mood with a single bout of dynamic taekwondo exercise in college-age students<br><br>11. Suicidal ideation among Bangladeshi university students early during the covid-19 pandemic: prevalence estimates and correlates<br><br>12. Association between sedentary behavior and depression among Japanese medical students during the covid-19 pandemic: a cross-sectional online survey<br><br>13. Mental health and physical activity in health-related university students during the covid-19 pandemic<br><br>14. The relationship between physical activity and anxiety in college students: exploring the mediating role of lifestyle habits and dietary nutrition<br><br>15. Exploring the interconnections of anxiety, depression, sleep problems and health-promoting lifestyles among Chinese university students: a comprehensive network approach<br><br>16. Relationship between social support and depressive symptoms in collegiate student athletes

	17. Incidence and risk factors of depressive symptoms in Chinese college students<br><br>18. Is there more to yoga than exercise?<br><br>19. Physical activity and depression among Korean female college students due to covid-19<br><br>20. Effects of physical activity on anxiety levels in college students: mediating role of emotion regulation<br><br>21. Research on the effect of basketball on mental rehabilitation of depressed patients<br><br>22. Impact of covid-19 on the life of higher-education students in Istanbul: relationship between social support, health-risk behaviors, and mental/academic well-being<br><br>23. Examination of the eating behaviours and depression states of the university students who stay at home during the coronavirus pandemic in terms of different variables<br><br>24. Physical activity and mental health in Brazilian university students: an analysis in different sexes during the covid-19 pandemic<br><br>25. Physical activity and mental health in undergraduate students<br><br>26. Depression and lifestyle among university students: A one-year follow-up study<br><br>27. Pro-inflammatory cytokines as predictors of antidepressant effects of exercise in major depressive disorder<br><br>28. Health-related behaviors and symptoms of anxiety and depression in Spanish nursing students: an observational study<br><br>29. A linear periodized resistance training program is effective at reducing depressive symptoms but not anxiety in females: a pilot<br><br>30. Moderating effects of clock genes dna methylation on the relationship between physical activity trajectories and depressive symptoms among Chinese college students<br><br>31. Exercise reduces depression and inflammation but intensity matters<br><br>32. The relationship between the activity level, internet addiction, and depressive symptoms of university students during the coronavirus disease-2019 outbreak cross-sectional study<br><br>33. Association between the risk of major depression and low physical activity in Peruvian workers studying in universities

	34. Investigating the effects of physical activity counselling on depressive symptoms and physical activity in female undergraduate students with depression: a multiple baseline single-subject design<br><br>35. Influence of aerobic exercise on depression<br><br>36. Changes in physical activity patterns prior to and during the coronavirus pandemic and their association with mental well-being:a multi-country web-based study among higher education students<br><br>37. The effect of physical exercise on the anxiety of college students in the post-pandemic era: the mediating role of social support and proactive personality<br><br>38. The effects of a self-management treatment package on daily step count in university students with depressive symptoms<br><br>39. Effect of university students' sedentary behavior on stress, anxiety, and depression<br><br>40. Correlation of exercise participation, behavioral inhibition and activation systems, and depressive symptoms in college students<br><br>41. Higher modified beck depression inventory scores are associated with body, eating, and exercise amounts<br><br>42. The effects of meridian exercise on anxiety, depression, and self-esteem of female college students in Korea<br><br>43. Effects of team sports on anxiety, depression, perceived stress, and sleep quality in college students<br><br>44. Exercise and depressive and anxious symptoms: What is the nature of their interrelations?<br><br>45. Association of sedentary behavior with anxiety, depression, and suicide ideation in college students<br><br>46. Physical exercise ameliorates anxiety, depression and sleep quality in college students: experimental evidence from exercise intensity and frequency<br><br>47. Effect of the personality traits of healthy Japanese workers on depressive symptoms and social adaptation, and on the achievement rate of exercise therapy to prevent major depression<br><br>48. The effect of aerobic exercise on the mental health of college students<br><br>49. Relationship between physical activity and college students' life satisfaction: the chain mediating effect of psychological resilience and negative emotions<br><br>50. Enhancing mental health, well-being and active lifestyles of university students by means of physical activity and exercise research programs

	51. Effects of selected aerobic exercises on the depression and concentrations of plasma serotonin in the depressed female students aged 18 to 25<br><br>52. Physical activity in the treatment of depression in college students<br><br>53. Vigorous physical activity and depressive symptoms in college students<br><br>54. Relationships between physical activity, body image, bmi, depression and anxiety in Chinese college students during the covid-19 pandemic<br><br>55. Comparison of physical activity and depression levels in physiotherapy students with and without recommended home-based exercises<br><br>56. Physical exercise and chronic pain in university students<br><br>57. Chinese college students' physical-exercise behavior, negative emotions, and their correlation during the covid-19 outbreak<br><br>58. Protective effect of exercise against depression, anxiety, and stress among university students based on their level of academic performance<br><br>59. Influence of the covid-19 pandemic on quality of life, mental health, and level of physical activity in colombian university workers: a longitudinal study<br><br>60. Associations of physical activity, screen time with depression, anxiety and sleep quality among Chinese college freshmen<br><br>61. Undergraduate physical activity and depressive symptoms: a national study<br><br>62. Sedentary behavior and physical activity predicting depressive symptoms in adolescents beyond attributes of health-related physical fitness<br><br>63. Body image and compulsive exercise: are there associations with depression among university students?<br><br>64. The impact of exercise performance dissatisfaction and physical exercise on symptoms of depression among college students: a gender comparison<br><br>65. The relationship between physical activity and life satisfaction among university students in China: the mediating role of self-efficacy and resilience<br><br>66. Volume and social context of physical activity in association with mental health, anxiety and depression among youth<br><br>67. The relationship between university students' psychological resilience and anxiety levels and comparison in terms of physical activity levels gender and academic achievement<br><br>68. Internet addiction and depressive symptoms: a dose- response effect mediated by levels of physical activity

	69. The association between physical activity and prevalence of anxiety and depression in medical students during covid-19 pandemic: A cross-sectional study<br><br>70. The effect of 8-week taekwondo exercise on the response inhibition of college students with depression symptoms-evidence based on erps<br><br>71. The effects of different forms of single moderate intensity exercise on inhibitory function and immediate emotions in college students with depressive symptoms: evidence from erps<br><br>72. Effect of acute aerobic exercise with different intensity on depression score of college students with depressive symptoms: evidence from resting EEG parietal area<br><br>73. Fitness for body and mind: a study of online aerobic fitness combined with a digital psychological intervention to improve depression and enhance mental health in university students<br><br>74. Digital era fitness: the impact of online physical exercise combined with interoceptive training on depressive symptoms in university students<br><br>75. Study on the impact of exercise intervention on depression in university students<br><br>76. Association between sedentary behaviour and depression, stress and anxiety among medical school students in chennai, India<br><br>77. Effect of yoga in medical students to reduce the level of depression, anxiety, and stress: pilot study (goodbye stress with yoga gsy)<br><br>78. Social connectedness, self-esteem, and depression symptomatology among collegiate athletes versus nonathletes<br><br>79. Does the association between depressive symptomatology and physical activity depend on body image perception? A survey of students from seven universities in the UK
Incomplete outcome data ($n = 55$)	1. Neural correlates of physical activity moderate the association between problematic mobile phone use and psychological symptoms<br><br>2. The effect of physical fitness exercise on relieving psychological anxiety of college students<br><br>3. Association of sedentary behavior and physical activity with depression in sport university students<br><br>4. Evaluation of depression and its correlates in terms of demographics, eating habits, and exercises among university students: a multicenter cross-sectional analysis

	5. Exploring the relationship between physical activity and inhibitory function in college students with depressive symptoms through eeg<br>6. The effect of baduanjin exercise on the physical and mental health of college students<br>7. Algorithms to predict anxiety and depression among university students in China after analyzing lifestyles and sport habits<br>8. Depression as compared to level of physical activity and internet addiction among polish physiotherapy students during the covid-19 pandemic<br>9. Physical activity and depression among health and social science students at public sector university, dammam, ksa<br>10. The effects of an 8-week taekwondo exercise intervention on inhibitory control in university students with depressive symptoms demonstrated the following—evidence from behavior and erps<br>11. Interactive effects of sleep and physical activity on depression among rural university students in China<br>12. Associations between sugar-sweetened beverages consumption, duration of physical exercise, and depressive symptoms among Tibetan university students at high altitude<br>13. Combined effect of the smartphone addiction and physical activity on the depressive symptoms in secondary school students: a cross sectional study in Shanghai, China<br>14. Relationship of physical activity with anxiety and depression symptoms in Chinese college students during the covid-19 outbreak<br>15. Physical activity and mental health in sports university students during the covid-19 school confinement in Shanghai<br>16. Correlation between physical exercise levels, depressive symptoms and sleep quality in college students: evidence from electroencephalography<br>17. The relationship between physical activity and psychological sub-health among high altitude region tibetan college students<br>18. Triangulated evidence provides no support for bidirectional causal pathways between diet/physical activity and depression/anxiety<br>19. Investigating the feasibility of exergame on sleep and emotion among university students<br>20. Depression, anxiety, and stress in medical students in Peru: a cross-sectional study

	21. Associations between physical activity and reduced rates of hopelessness, depression, and suicidal behavior among college students<br><br>22. The moderating effect of physical activity in the relation between problematic mobile phone use and depression among university students<br><br>23. Depression symptoms related to undertaking regular physical activity in students of the faculty of physiotherapy<br><br>24. Ruminative self-focus in daily life: associations with daily activities and depressive symptoms<br><br>25. Physical activity, sedentary behaviour and symptoms of anxiety in post-secondary students: a cross-sectional study of two faculties<br><br>26. Effect of exercise intervention and rehabilitation on patients with depression<br><br>27. Nursing students and depressive symptomatology: an observational study in university of palermo<br><br>28. Substitutions of physical activity and sedentary behavior with negative emotions and sex difference among college students<br><br>29. First-year undergraduate students: depressed, distressed, and drained? Influence of depressive symptoms on markers of psychological well-being, sleep, and physical activity<br><br>30. Psychological distress in nursing students: relationship with screen time, diet and physical activity<br><br>31. Enhancing mental well-being of undergraduates: establishing cut-off values and analyzing substitutive effects of physical activity on depression regulation<br><br>32. The relationship between locomotive syndrome and depression in young Chinese college students<br><br>33. The impact of single sessions of aerobic exercise at varying intensities on depressive symptoms in college students: evidence from resting-state eeg in the parietal region<br><br>34. Correlation research on physical activity and executive function in female college students with subclinical depression<br><br>35. Latent profile analysis of depressive symptoms in college students and its relationship with physical activity<br><br>36. Depressive symptoms associated with loneliness and physical activities among graduate university students in Bangladesh: findings from a cross-sectional pilot study

	37. Influence of moderate-to-high intensity physical activity on depression levels: a study based on a health survey of Chinese university students<br><br>38. Associations between screen time, physical activity, and depressive symptoms during the 2019 coronavirus disease (covid-19) outbreak among Chinese college students<br><br>39. Canonical correlation analysis of depression and anxiety symptoms among college students and their relationship with physical activity<br><br>40. The effect of college students' physical activity level on depression and personal relationships<br><br>41. Exploring the relationship between physical activity and inhibitory function in college students with depressive symptoms through eeg<br><br>42. Prevalence and factors associated with depression and anxiety among first-year university students in Bangladesh: a cross-sectional study<br><br>43. Inadequate mental health literacy and insufficient physical activity potentially increase the risks of anxiety and depressive symptoms in Chinese college students<br><br>44. The independent, joint, and additive associations of physical activity and self-compassion on depression symptoms among Chinese college students<br><br>45. Association between 24-hour movement behavior and depression in college students: a compositional data analysis<br><br>46. The associations between body dissatisfaction, exercise intensity, sleep quality, and depression in university students in southern China<br><br>47. Intensity of physical activity and depressive symptoms in college students: fitness improvement tactics in youth (fityou) Project<br><br>48. Participation in higher intensity physical activity predicts lower depressive symptom incidence in college students<br><br>49. Physical activity and mental health. Is achieving the physical activity guidelines associated with less depressive symptoms among undergraduates at the university of turku, finland?<br><br>50. Does the association between depressive symptomatology and physical activity depend on body image perception? A survey of students from seven universities in the UK<br><br>51. The effect of physical exercise of different intensities on the mental health of college students<br><br>52. Relationship of physical activity and sleep with depression in college students

	53. Exercise and pharmacological treatment of depressive symptoms in patients with coronary heart disease<br>54. The effect of replacing sedentary behavior with different intensities of physical activity on depression and anxiety in Chinese university students: an isotemporal substitution model<br>55. The role of self-esteem and depression in the relationship between physical activity and academic procrastination among Chinese undergraduate students: a serial mediation model
Full-text unavailable (*n* = 6)	1. Runningtherapie, stress, depression-ein uebungszentrierter ansatz in der integrativen leib- und bewegungsorientierten psychotherapie. Running therapy, stress, depression: an exercise-centered approach in integrative body and movement therapy<br>2. Self-regulation of physical exercise on mental status of patients with depression<br>3. Correlations between mental health, physical activity, and body composition in American college students after the covid-19 pandemic lockdown<br>4. The level of depression among university students with the role of physical activity and daytime sleepiness<br>5. Examining anxiety, sleep quality, and physical activity as predictors of depression among university students from Saudi Arabia during the second wave of the covid-19 pandemic<br>6. An exploratory examination of the relationship between symptoms of depression and exercise addiction among undergraduate recreational exercisers
Non-English publications (*n* = 4)	1. Association between changes in physical activity and<br>comorbid symptoms of anxiety and depression in college students<br>2. Relationships among lifestyle, depression, anxiety, and reproductive health in female university students<br>3. Health qigong wuqinxi improves hydrogen proton magnetic resonance spectra in prefrontal cortex and hippocampus in college students with mild depression<br>4. A chain-mediated model of the effect of physical exercise on loneliness

Table S3. Characteristics of studies included in this meta-analysis.

Study	Country	Sample size (male/female)	Timing of data collection	Academic discipline	PA measurement	DQ measurement	r
Alcalde et al., 2022 [60]	Peru	321 (205/116)	During COVID-19	-	IPAQ	PHQ-9	−0.172
Cecchini et al., 2019 [65]	Spain	353 (176/177)	Pre-COVID-19	-	IPAQ	A six-item self-report scale	−0.15
Coughenour et al., 2021 [61]	USA	194 (53/141)	During COVID-19	-	PA-I	PHQ-9	−0.151
Fu et al., 2023 [35]	China	478	Post-COVID-19	Physical Education	PARS-3	SDS	−0.621
Gaia et al., 2024 [54]	Iran	100 (53/47)	Post-COVID-19	-	IPAQ	BDI	−0.433
Gerber et al., 2014 [20]	Switzerland	451 (171/280)	Pre-COVID-19	Medicine, Exercise and Health Sciences	Office in motion questionnaire	Depression scale	−0.03

Ghassab-Abdollahi et al., 2020 [55]	Iran	186	Pre-COVID-19	Medicine	International PA questionnaire	Beck depression inventory-2	−0.573
Han et al., 2023 [36]	China	251 (145/106)	During COVID-19	-	IPAQ-SF	SDS	−0.216
Herbert et al., 2020 [66]	Germany	185 (28/157)	Pre-COVID-19	-	GPAQ	BDI-II	−0.22
Huang et al., 2024 [37]	China	326 (166/160)	Post-COVID-19	Physical Education	Physical activity rating scale	DASS-21	−0.379
Kardangusheva et al., 2022 [67]	Russia	425 (114/311)	During COVID-19	Medicine	-	HADS	−0.17
Kim et al., 2021 [56]	South Korea	525 (259/266)	Pre-COVID-19	-	IPAQ	BDI	−0.154
Li et al., 2023 [39]	China	2606 (1740/866)	During COVID-19	Physical Education	IPAQ	BDI-II	−0.107
Li et al., 2024 [38]	China	78 (0/78)	During COVID-19	-	ActiGraph GT3X accelerometer	HADS	−0.64

Lin et al., 2022 [40]	China	605 (320/285)	Pre-COVID-19	-	Fox	Development of the Taiwanese depression questionnaire	−0.34
Liu et al., 2023 [41]	China	1290 (650/640)	During COVID-19	Physical Education	Questionnaire of college students	Depression scale	−0.399
Ofili et al., 2024-1 [69]	Nigeria	418	During COVID-19	-	IPAQ-SF	PHQ-9	−0.9
Ofili et al., 2024-2 [69]	Nigeria	418	During COVID-19	-	-	BDI-II	−0.54
Ofili et al., 2024-3 [69]	Nigeria	418	During COVID-19	-	PARS-3	DASS	−0.55
Ofili et al., 2024-4 [69]	Nigeria	418	During COVID-19	-	Godin-shephard leisure time physical activity questionnaire	PHQ-9	−0.68
Pelzer et al., 2022 [68]	Germany	220 (64/156)	Pre-COVID-19	Medicine	Steps per day	PHQ-9	−0.3
Qin et al., 2024 [42]	China	5341 (1713/3628)	During COVID-19	-	IPAQ	BDI-II	−0.162

Shen et al., 2025-1 [43]	China	488 (132/356)	During COVID-19	Economics, Management Sciences, Law Literature, Science, Engineering, Arts	IPAQ	Beck inventory	−0.109
Shen et al., 2025-2 [43]	China	488 (132/356)	During COVID-19		CDC’s youth risk behavior survey	CES-D	−0.045
Shen et al., 2025-3 [43]	China	488 (132/356)	During COVID-19		PARS-3	SDS	−0.035
Shen et al., 2025-4 [43]	China	488 (132/356)	During COVID-19		IPAQ	DASS-21	−0.001
Shen et al., 2025-5 [43]	China	488 (132/356)	During COVID-19		Single self-reported item	DASS-21	0.25
Shen et al., 2025-6 [43]	China	488 (132/356)	During COVID-19		PSPP	SDS	0.34
Shen et al., 2025-7 [43]	China	488 (132/356)	During COVID-19		PARS-3	SDS	0.02
Shen et al., 2025-8 [43]	China	488 (132/356)	During COVID-19		PARS-3	PHQ-9	−0.018
Shen et al., 2025-9 [43]	China	488 (132/356)	During COVID-19		Level of daily exercise	SDS	−0.003

Shimamoto et al., 2021 [21]	Japan	85 (52/33)	Pre-COVID-19	Literature, Law, Economics, Foreign Languages, Engineering, Medicine	-	PHQ-9	0.39
Song et al., 2022 [57]	South Korea	260 (0/260)	During COVID-19	-	LTPA questionnaire	PHQ-9	−0.11
Souza et al., 2021 [62]	Brazil	180 (117/63)	During COVID-19	Medicine, Physical Education	PARS	CFI	−0.23
Taliaferro et al., 2010 [18]	USA	450 (117/333)	Pre-COVID-19	-	PARS-3	DASS-21	−0.17
Tang et al., 2022 [44]	China	479 (293/186)	During COVID-19	-	Physical exercise intensity scale for college students	4-point Likert scale ranging from	−0.198
Torales et al., 2024 [63]	Paraguay	317 (97/220)	Post-COVID-19	Agricultural Sciences, Natural Sciences, Social Sciences, Health	IPAQ-SF<br>PSQI	PHQ-9	−0.167

				Sciences, Biology, Chemistry, Engineering, Mathematics, Informatics, Physics			
Vally et al., 2021-1 [59]	Total	1322	During COVID-19	-	International physical activity questionnaire short form	BDI-II	−0.08
Vally et al., 2021-2 [59]	Egypt	1036	During COVID-19	-	PA measurement	DQ measurement	−0.09
Vally et al., 2021-3 [59]	UAE	286	During COVID-19	-	IPAQ	PHQ-9	−0.06
Wang et al., 2024 [45]	China	766 (314/452)	During COVID-19	-	IPAQ	A six-item self-report scale	−0.531
Wei et al., 2024 [46]	China	579 (132/447)	During COVID-19	-	PA-I	PHQ-9	−0.181

Yang et al., 2022 [47]	China	586 (334/252)	During COVID-19	-	PARS-3	SDS	−0.417
Yano et al., 2018 [58]	Japan	275 (135/140)	Pre-COVID-19	-	IPAQ	BDI	−0.33
Yates et al., 2020 [64]	USA	9	During COVID-19	-	Office in motion questionnaire	Depression scale	−0.656
Ye et al., 2024-1 [48]	China	305 (305/0)	Post-COVID-19	-	International PA questionnaire	Beck depression inventory-2	−0.284
Ye et al., 2024-2 [48]	China	305 (305/0)	Post-COVID-19		IPAQ-SF	SDS	−0.249
Yu et al., 2025 [49]	China	2537	Post-COVID-19	Physical Education, Accounting and Finance	GPAQ	BDI-II	−0.063
Yue et al., 2022 [50]	China	2217 (702/1515)	During COVID-19	Medicine	Physical activity rating scale	DASS-21	−0.052
Zhang et al., 2022 [52]	China	1097 (440/657)	During COVID-19	-	-	HADS	−0.221

Zhang et al., 2024 [53]	China	1793 (653/1140)	Pre-COVID-19	-	IPAQ	BDI	−0.2
Zhu et al., 2023 [51]	China	442	During COVID-19	-	IPAQ	BDI-II	−0.175

Abbreviations: DQ, Depression Questionnaire; PA, Physical Activity; r, correlation; PHQ-9, Patient Health Questionnaire-9; IPAQ, International Physical Activity Questionnaire; PA-I, Physical Activity-Inform; SDS, Self - rating Depression Scale; PARS-3, Physical Activity Rating Scale-3; BDI, Beck Depression Inventory; IPAQ-SF, International Physical Activity Questionnaire-Short Form; BDI-II, Beck Depression Inventory - II; GPAQ, Global Physical Activity Questionnaire; DASS-2, Depression Anxiety Stress Scales-21; HADS, Hospital Anxiety and Depression Scale; CES-D, Center for Epidemiological Studies-Depression; LTPA Questionnaire, Leisure Time Physical Activity Questionnaire; CFI, Cognitive Flexibility Inventory; PARS, Physical Activity Rating Scale; DASS-21, Depression Anxiety Stress Scales-21; PARS-3, Physical Activity Rating Scale-3.

Table S4. Details of the scoring criteria in the JBI appraisal checklist.

Study	Joanna Briggs Institute appraisal checklist items										Total score (%)	Overall risk of bias
	1	2	3	4	5	6	7	8	9	10		
Alcalde et al., 2022 [60]	2	0	1	2	2	0	2	2	2	2	15 (75)	Low
Cecchini et al., 2019 [65]	2	1	1	2	2	0	2	2	2	1	15 (75)	Low
Coughenour et al., 2021 [61]	2	0	1	2	2	0	2	2	2	1	14 (70)	Low
Fu et al., 2023 [35]	2	2	2	2	2	0	2	2	2	1	17 (85)	Low
Gaia et al., 2024 [54]	2	2	2	2	2	0	2	2	2	1	17 (85)	Low
Gerber et al., 2014 [20]	2	0	1	2	2	0	2	2	2	1	14 (70)	Low
Ghassab-Abdollahi et al., 2020 [55]	2	2	2	2	2	1	2	2	2	1	18 (90)	Low
Han et al., 2023 [36]	2	0	1	2	2	0	2	2	2	2	15 (75)	Low

Herbert et al., 2020 [66]	2	0	2	2	2	0	2	2	2	1	15 (75)	Low
Huang et al., 2024 [37]	2	0	1	2	2	0	2	2	2	2	15 (75)	Low
Kardangusheva et al., 2022 [67]	2	1	1	2	2	1	1	2	2	2	16 (80)	Low
Kim et al., 2021 [56]	2	0	1	2	2	0	2	2	2	1	14 (70)	Low
Li et al., 2023 [39]	2	2	1	1	2	0	2	2	1	2	15 (75)	Low
Li et al., 2024 [38]	2	0	2	2	2	0	2	2	2	2	16 (80)	Low
Lin et al., 2022 [40]	2	2	1	1	2	0	2	2	1	2	15 (75)	Low
Liu et al., 2023 [41]	2	2	1	1	2	0	2	2	1	2	15 (75)	Low
Ofili et al., 2024 [69]	2	1	2	2	2	0	2	2	2	2	17 (85)	Low
Pelzer et al., 2022 [68]	2	2	0	1	2	0	2	2	2	1	14 (70)	Low
Qin et al., 2024 [42]	2	2	1	1	2	0	2	2	1	2	15 (75)	Low
Shen et al., 2023 [43]	2	1	1	2	2	1	2	2	2	2	17 (85)	Low
Shimamoto et al., 2021 [21]	2	1	1	2	2	0	2	2	2	2	16 (80)	Low
Song et al., 2022 [57]	2	0	1	2	2	0	2	2	2	2	15 (75)	Low

Souza et al., 2021 [62]	2	1	1	2	2	0	2	2	2	2	16 (80)	Low
Taliaferro et al., 2010 [18]	2	1	2	2	2	1	2	2	2	1	17 (85)	Low
Tang et al., 2022 [44]	2	1	2	2	2	1	2	2	2	1	17 (85)	Low
Torales et al., 2024 [63]	2	1	2	2	2	1	2	2	2	2	18 (90)	Low
Vally et al., 2021 [59]	2	1	1	2	2	1	2	2	2	2	17 (85)	Low
Wang et al., 2024 [45]	2	1	2	2	2	1	2	2	2	2	18 (90)	Low
Wei et al., 2024 [46]	2	1	1	2	2	1	2	2	2	2	17 (85)	Low
Yang et al., 2022 [47]	2	1	1	2	2	0	2	2	2	1	15 (75)	Low
Yano et al., 2018 [58]	2	1	1	2	2	1	2	2	2	2	17 (85)	Low
Yates et al., 2019 [64]	2	1	2	2	2	1	2	2	2	2	18 (90)	Low
Ye et al., 2024 [48]	2	1	1	2	2	0	2	2	2	2	16 (80)	Low
Yu et al., 2024 [49]	2	1	0	2	2	0	2	2	1	2	14 (70)	Low
Yue et al., 2022 [50]	2	1	0	2	2	0	2	2	1	2	14 (70)	Low
Zhang et al., 2022 [52]	2	1	0	2	2	0	2	2	1	2	14 (70)	Low
Zhang et al., 2024 [53]	2	2	0	1	2	1	2	2	2	1	15 (75)	Low

Zhu et al., 2023 [51]	2	1	2	2	2	1	2	2	2	1	17 (85)	Low
